# Eye-tracking indices of impaired encoding of visual short-term memory in familial Alzheimer’s disease

**DOI:** 10.1038/s41598-021-88001-4

**Published:** 2021-04-22

**Authors:** Ivanna M. Pavisic, Yoni Pertzov, Jennifer M. Nicholas, Antoinette O’Connor, Kirsty Lu, Keir X. X. Yong, Masud Husain, Nick C. Fox, Sebastian J. Crutch

**Affiliations:** 1 Department of Neurodegenerative Diseases, Dementia Research Centre, UCL Queen Square Institute of Neurology, London, WC1N 3BG UK; 2 UK Dementia Research Institute at University College London, London, UK; 3 Department of Psychology, The Hebrew University of Jerusalem, Jerusalem, Israel; 4 Department of Medial Statistics, London School of Hygiene and Tropical Medicine, London, UK; 5 Nuffield Department of Clinical Neuroscience, University of Oxford, Oxford, UK; 6 Department of Experimental Psychology, University of Oxford, Oxford, UK

**Keywords:** Cognitive neuroscience, Neuroscience, Neurology, Diseases

## Abstract

The basis of visual short-term memory (VSTM) impairments in preclinical Alzheimer’s disease (AD) remains unclear. Research suggests that eye movements may serve as indirect surrogates to investigate VSTM. Yet, investigations in preclinical populations are lacking. Fifty-two individuals from a familial Alzheimer’s disease (FAD) cohort (9 symptomatic carriers, 17 presymptomatic carriers and 26 controls) completed the “Object-localisation” VSTM task while an eye-tracker recorded eye movements during the stimulus presentation. VSTM function and oculomotor performance were compared between groups and their association during encoding investigated. Compared to controls, symptomatic FAD carriers showed eye movement patterns suggestive of an ineffective encoding and presymptomatic FAD carriers within 6 years of their expected age at symptom onset, were more reliant on the stimuli fixation time to achieve accuracy in the localisation of the target. Consequently, for shorter fixation times on the stimuli, presymptomatic carriers were less accurate at localising the target than controls. By contrast, the only deficits detected on behavioural VSTM function was in symptomatic individuals. Our findings provide novel evidence that encoding processes may be vulnerable and weakened in presymptomatic FAD carriers, most prominently for spatial memory, suggesting a possible explanation for the subtle VSTM impairments observed in the preclinical stages of AD.

## Introduction

Deficits of episodic memory in Alzheimer’s disease (AD) are well characterised but, until recently, short-term memory (STM) function has attracted far less attention. The ability to hold onto information over short periods of few seconds has a pivotal role in almost every cognitive task. In the last decade, one important line of research has provided evidence that the ability to bind the different features of an object together might be critically affected in AD. In their pioneering studies, Parra and colleagues reported that the conjunctive binding—the integration of features within an object such as colour and shape or colour and colour—was selectively disrupted in AD in visual STM (VSTM)^1^. These investigations employed a *change detection paradigm* which measures VSTM *capacity* (the number of items an individual can remember over short durations) and relies on a binary recall accuracy response (either something is remembered or not)^1^. Somewhat complementary, is relational binding—the association of an object’s identity to other ‘independent’ features such as its location—often assessed through *delayed-reproduction tasks* which capture the *resolution* with which items are retained in memory^2^ using a *continuous* analogue response space. VSTM impairments, evaluated either through change detection or delayed-reproduction tasks, have been reported in preclinical AD populations including presymptomatic familial Alzheimer’s disease [FAD, an autosomal dominantly inherited condition caused by mutations in either presenilin 1 (*PSEN1*), presenilin 2 (*PSEN2*) or amyloid precursor protein (*APP*)]^3–5^. Whether one type of binding is best suited to study AD is open to debate. Some argue that the resistance of conjunctive binding to healthy ageing favors it over relational binding^6,7^ and others propose the sensitivity to ageing translates into a high predictive power when comparing patients’ performance to that of *age-matched* controls^8^. Regardless, both approaches have often been in line with resources models of working memory, suggesting that recall variability increases gradually and continuously with increasing set size (i.e. the precision of one object comes as the cost of other stimuli held in memory)^9,10^. The *precision* of recall has been reported to be more sensitive than conventional span measures which only index the *number* of items held in memory^11^ yet until recently, measuring cognitive ability focused on accuracy and speed. Over the past years, eye movement investigations have emerged as useful and sensitive tools for measuring subtle cognitive processes (e.g. attention^12^, executive control^13^ and working memory^14^), even in the absence of conscious recollection^15^ (e.g. gaze duration to an object in a scene is longer when it is replaced, even when participants fail to explicitly detect the change^16^). Importantly, eye-tracking may offer greater insight into the cognitive processes underlying behavioural outcomes. For instance, a study in mild AD showed that reduction of fixation duration during the encoding of the stimuli, led to poor performance^17^.

The nature of human visual processing is such that one region of the visual scene is sampled at a time, by directing the high-acuity foveal portion of the retina to selected regions^15^. Such patterns of exploration, captured by gaze position across time, appear to be particularly influenced by two types of factors: the physical properties of the elements (‘bottom-up’) and the contextual information available (‘top-down’)^18^. A prevalent view of such sequential sampling is that at every fixation, the oculomotor system faces competition between exploring different aspects of an object or scene *vs* maintaining fixation to allow for in-depth processing (the ‘exploration–exploitation dilemma’)^19^. The ‘linear approach to threshold explaining space and time’ (LATEST) model of gaze deployment, claims that each decision to move the eyes is “*an evaluation of the relative benefit expected from moving the eyes to a new location compared with that expected by continuing to fixate the current target*”^20^. Theoretically, the eyes move when the evidence that favours shifting to a new location outweighs that favouring to remain at the present location^20^. Reports have also proposed eye movements act as indirect surrogates of VSTM^17,21^ as better recall or ‘stronger memories’ are associated with image regions that attract more fixations during encoding^15,22^—provided that the fixation duration is sufficient for encoding (usually ≥ 150 ms^23^ though this is dependent on the stimuli). Taken together, these investigations propose eye movements as suitable candidates to study memory processes.

To understand the role and validity of VSTM delayed-reproduction tasks as preclinical cognitive markers, it is necessary to determine whether VSTM deficits may arise from alterations in: (i) correctly *maintaining* the features of an item; (ii) variability in the ability to access the memory (*retrieval*) or (iii) correctly *encoding* the stimuli in the first place. Studying eye movements may help unveil the source of impairment. For instance, if individuals carrying a genetic mutation for FAD, have a different eye movement pattern than controls during the initial presentation of the stimuli, could this be an indication of an encoding impairment? Moreover, how do different visual search strategies relate to the accuracy of task performance?

This paper, will evaluate the relationship between eye movements and VSTM function (specifically, the recall for the object’s identity and its location and, in doing so, also measure relational binding accuracy), during encoding. Encoding represents the first stage in the memory formation process and here, will be indexed indirectly by the overall time spent fixating the stimulus. Based on the study of eye movements in mild AD previously mentioned^17^ and reports of high mean diffusivity in the precuneus of FAD presymptomatic mutation carriers (PMCs)^24^ (a region important for mental and visuo-spatial imagery and closely related to working memory^25^), we hypothesize that encoding might be particularly affected in presymptomatic FAD individuals.

To our knowledge, no other studies have evaluated the relationship between eye movements and memory deficits, over short or long durations, in FAD.

**Figure 1:**
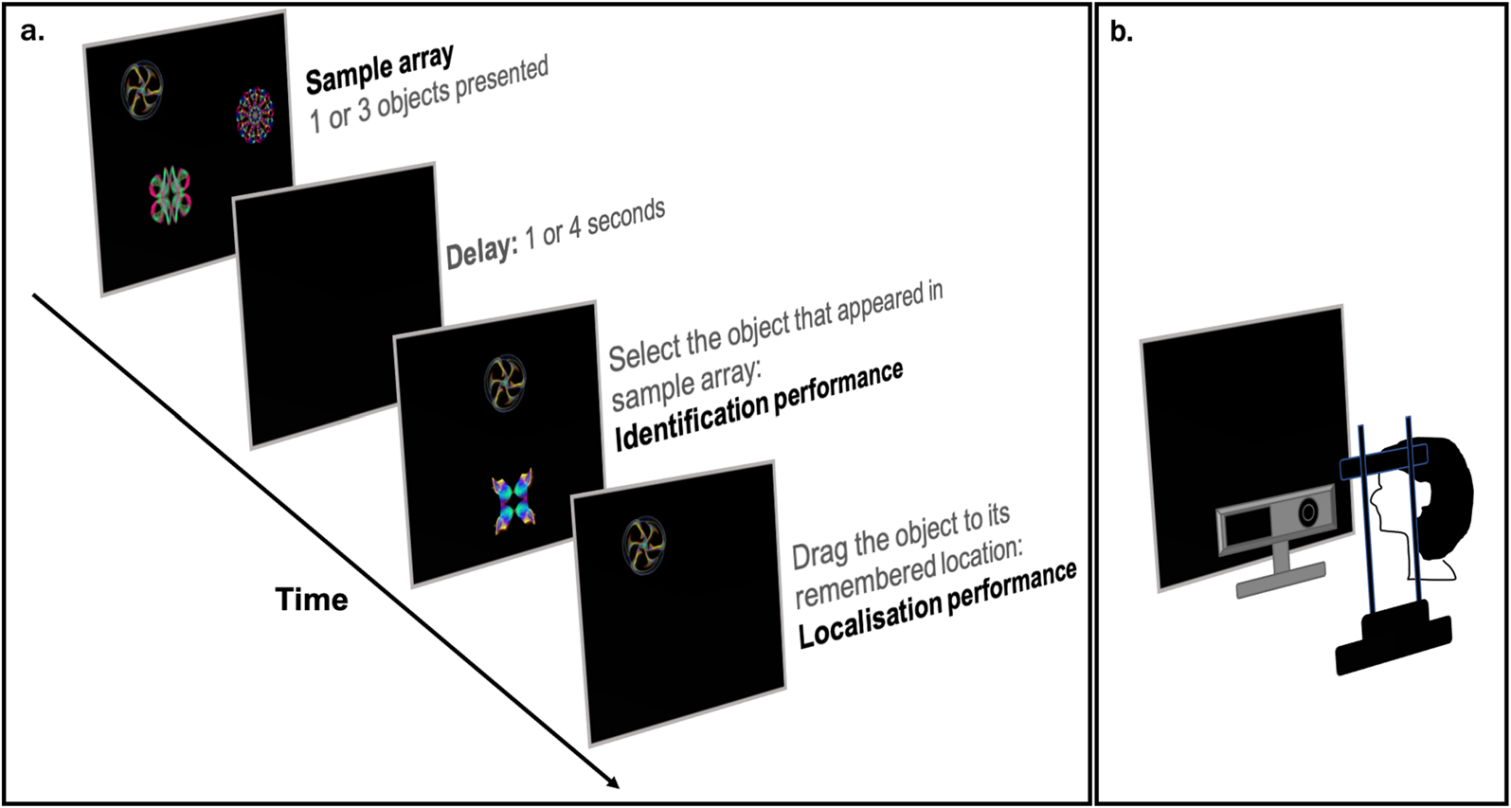
(**a**) Schematic of “Object-localisation” task^21^ with identification and localisation measures described [adapted from Liang et al. (2016) under the terms of the Creative Commons Attribution License (CC BY)]. (**b**) The eye-tracking setup used throughout the experiment.

Overall, 52 participants (26 carriers of mutations 9 of which had progressive cognitive symptoms in *PSEN1* or *APP* and 26 healthy controls), completed the “Object-localisation” VSTM task. A schematic of the task is presented in Fig. 1. In short, in each trial participants viewed a sample array of 1 or 3 fractal objects (to test for the effect of cognitive load) for a period of 1 and 3 s respectively, each randomly located on the screen and were asked to remember both the objects and their locations. A blank screen was then displayed for a 1 or 4 s duration (to test for the effect of retention duration or delay interval), followed by a test array in which two fractals appeared along the vertical meridian. One of these was in the previous memory array (the target fractal) whereas the other one was a foil (distractor). Participants were required to touch the fractal they remember from the memory array (identification accuracy) and drag it on the touch screen to its location (localisation error). The design consisted of 10 trials with 1 fractal and 40 trials with 3 fractals and a balanced number of trials with 1 or 3 fractals and 1- or 4-s delay between memory and test arrays. In line with previous reports (e.g. Ref.^21^) cognitive demand is highest in high-load and long-delay conditions as memory recall precision decreases when resources are distributed among objects (vs one object) and following a long-delay (vs a short-delay).

Eye movements were recorded during the sample array at 1000 Hz using a desktop-mounted infrared video-based eye tracker (Eyelink 1000Plus; SR Research). Participants sat in front of the computer screen resting their head on a chin rest in order to provide stability and maintain a fixed viewing distance. The eye-tracking camera did not obstruct the computer screen but was placed below it. Before the experiment began, the tester ensured the participant was able to reach the computer touch-screen comfortably without obstructing the eye-tracking camera in the process.

A region of interest (ROI) of 8.4 deg in diameter was set for each fractal (the fractal diameter was 5.7 deg wide which resulted in a 1.35 deg ROI border). Eye-tracking investigations were restricted to the 3-item trials given the focus on visual exploration strategies (see “Methods” section for details on the eye-tracking metrics).

As the aim of the current paper was to investigate eye-movement contributions for the encoding of perceptual and spatial features, results were focused on the two memory measures of task performance measuring these aspects: identification accuracy (i.e. proportion of trials where the correct object was chosen) and localisation error (i.e. the distance in visual angle between the centre of the target object once placed in its remembered location and its original location in the memory array—only correctly identified objects).

## Results

### Participant characteristics

Demographics and traditional neuropsychology characteristics of participants is shown in Table 1. Individuals were classified based on mutation status, presence or absence of clinical symptoms and their proximity to expected age at symptom onset (EYO) into the following groups: control, ‘early’ PMC, ‘late’ PMC or symptomatic mutation carrier (SMC). One late PMC participant was excluded from all analysis due to intermittent signal loss throughout the experiment (see Table 1 for demographics information and “Methods” section for a more detailed description of participants). There was no difference in the distribution of sexes in the sample as a whole (χ^2^ = 5.23, *p* = 0.169) or between each patient group and controls (early PMCs: χ^2^ = 0.44, *p* = 0.676; late PMCs: χ^2^ = 0.22, *p* = 0.712; SMC: χ^2^ = 3.37, *p* = 0.121).

**Table 1:** Participant demographics and neuropsychology: mean and SD unless stated otherwise.

	Controls (N = 26)	Early PMCs (N = 7)	Late PMCs (N = 9)	SMCs (N = 9)
**Demographics**				
Gender (male: female)	11: 15	2: 5	3: 6	7: 2
Age (years)	38.5 (11.8)	38.1 (4.7)	41.3 (7.6)	50.0 (10.4)**
MMSE	29.9 (0.3)	29.4 (0.5)	29.8 (0.4)	25.0 (2.6)**
NART^a^	29.7 (8.0)	26.9 (11.1)	31.4 (3.5)	30.9 (10.2)
Education (years)	16.2 (2.1)	16.3 (2.4)	14.4 (2.7)*	15.4 (2.0)
CDR (global)	0.0 (0.0)	0.0 (0.0)	0.0 (0.0)	1.7 (2.1)**
Anxiety^b^	6.9 (4.0)	9.0 (3.5)	7.0 (4.5)	4.1 (2.0)*
Depression^b^	2.0 (3.0)	3.9 (3.8)*	2.0 (2.8)	2.3 (2.8)
SCD: MyCog^c^	1.5 (2.7)	5.1 (7.1)*	3.6 (3.2)	15.9 (5.4)**
AD8^d^	0.5 (1.9)	0.5 (0.8)	0.0 (0.0)	5.3 (1.7)**
**Neuropsychology**				
Verbal IQ^e^	101.6 (8.5)	102.0 (12.2)	105.9 (14.1)	97.2 (14.6)
Performance IQ^e^	115.9 (13.7)	112.1 (15.2)	114.8 (12.0)	92.0 (14.1)**
Arithmetic total/24^c^	11.9 (7.9)	9.7 (4.9)	12.9 (4.7)	7.3 (6.8)
RMT faces^e^	45.4 (3.6)	44.3 (4.2)	45.1 (2.8)	37.7 (7.3)**
RMT words^e^	48.9 (1.6)	50. 0 (0.0)	47.0 (3.2)	34.4 (5.8)**
Digit span forwards/8^e^	7.2 (0.7)	6.9 (0.9)	7.3 (0.7)	6.2 (1.3)*
Digit span backwards/7^e^	4.9 (1.2)	4.9 (1.2)	5.2 (0.8)	4.3 (1.6)
BPVS^f^	140. 8 (8.0)	136.9 (14.2)	143.4 (3.9)	140.7 (9.7)
Verbal fluency^e^	15.3 (5.1)	16.0 (2.0)	16.3 (4.2)	13.3 (6.1)
Category Fluency^e^	24.5 (6.2)	22.3 (3.4)	24.1 (4.9)	15.9 (5.9)**
GNT/30^f^	19.2 (4.7)	18.3 (5.8)	22.9 (1.6)	18.7 (5.9)
VOSP OD/20^f^	18.6 (1.1)	17.7 (2.4)	19.1 (1.0)	17.1 (2.3)*
Stroop ink time (s)^f^	48.4 (11.3)	51.4 (11.4)	48.3 (10.0)	99.3 (43.0)**
Camden PAL^g^	19.8 (4.4)	18.6 (3.2)	19.9 (5.0)	6.7 (4.5)**
Digit symbol^e^	65.9 (11.8)	65.6 (4.6)	66.7 (11.7)	31.1 (12.5)**
Spatial forwards/9^f^	6.4 (0.8)	5.4 (1.3)	5.9 (0.9)	4.1 (1.5)**
Spatial backwards/9^f^	5.8 (1.0)	4.9 (1.6)	5.1 (0.9)	3.4 (1.5)**
Trails A time (s)^e^	24.9 (7.2)	24.6 (9.9)	21.0 (4.9)	53.3 (37.2)**
Trails B time (s)^g^	54.2 (16.5)	58.1 (22.6)	46.3 (5.6)	153.6 (90.3)**

*SD* standard deviation, *PMC* presymptomatic mutation carrier, *SMC* symptomatic mutation carrier, *MMSE* mini-mental state examination, *CDR* clinical dementia rating scale; Anxiety and depression from the *HADS* hospital anxiety and depression scale, *SCD* subjective cognitive decline, *IQ* intelligent quotient, *RMT* recognition memory test, *BPVS* British Picture Vocabulary Scale, *GNT* graded naming test, *VOSP OD* Visual Object and Space Perception Battery: Object Decision, *NART* National Adult Reading Test, *Camden PAL* Camden paired associated learning; Digit spans forwards and backwards from the WMS-R** = **Wechsler Memory Scale. ^a^n = 48; ^b^n = 38; ^c^n = 39; ^d^n = 37; ^e^n = 44; ^f^n = 43; ^g^n = 42. Bold = significant; *significant at *p* < 0.05; **significant at *p* < 0.01.

There was a significant difference between groups in: age (*F*(3,47) = 3.00, *p* = 0.040, η^2^_p_ = 0.16); MMSE scores (χ^2^(3) = 20.87, *p* < 0.001); CDR score (χ^2^(3) = 21.00, *p* < 0.001); anxiety reports (χ^2^(3) = 8.37, *p* = 0.039) and the individuals’ and informant’ perception of the participant’s cognitive decline [MyCog scale: (χ^2^(3) = 20.58, *p* < 0.001) and AD8 scale: χ^2^(3) = 16.57, *p* < 0.001)]. Overall, no differences in education level (χ^2^(3) = 0.24, *p* = 0.244) or depression reports (χ^2^(3) = 3.16, *p* = 0.367) were observed. Post-hoc pairwise comparisons revealed the significant differences lied between SMCs and controls in: age (*p* = 0.006); MMSE (*p* < 0.001); anxiety reports (*p* = 0.016) and symptoms of subjective cognitive decline (MyCog and AD8, both *p* < 0.001). While both PMC groups were well-matched for age compared to controls, early PMCs reported higher MyCog (*p* = 0.023) and depression scores (*p* = 0.034) and late PMCs had slightly lower education levels (*p* = 0.025).

Neuropsychology differences were observed in: non-verbal reasoning [performance IQ: *F*(3,40) = 6.76, *p* < 0.001, η^2^_p_ = 0.34]; recognition memory [RMT for faces: χ^2^(3) = 9.18, *p* = 0.027 and words: χ^2^(3) = 24.67, *p* < 0.001]; category fluency [*F*(3,40) = 5.36, *p* = 0.003, η^2^_p_ = 0.28]; executive function [Stroop: χ^2^(3) = 16.84, *p* < 0.001, Trails A and B both *p* < 0.001]; associative learning [Camden paired associated learning-Camden PAL: χ^2^(3) = 14.38, *p* = 0.002]; processing speed [digit symbol: χ^2^(3) = 20.54, *p* < 0.001]; spatial STM [spatial digit span forwards; *F*(3,39) = 8.64, *p* < 0.001, η^2^_p_ = 0.40] and spatial working memory [spatial digit span backwards: *F*(3,39) = 7.58, *p* < 0.001, η^2^_p_ = 0.37]. Consistent with other reports (e.g. Ref.^26^), post-hoc pairwise comparisons revealed significant differences only between SMCs and controls: performance IQ: *p* < 0.001; digit span forwards: *p* = 0.016; object perception: *p* = 0.036; RMT faces: *p* = 0.002; RMT words; Stroop; spatial digit span forwards and backwards; Camden PAL and digit symbol (all *p* < 0.001).

### Behavioural metrics of task performance

Behavioural performance on the “Object-localisation” VSTM task was compared between groups. Localisation error for each trial was log-transformed and analysed using a linear regression model and analysis of object identity used a logistic regression model (see “Methods” for more details on statistical analysis). Consistent with previous studies using this task^21,27^, across the sample as a whole, identification and localisation performance were significantly worse with higher memory load (3- vs 1-items) (identification: Odd Ratio for correct response (OR) 0.19 [95% CI 0.10, 0.35], *p* < 0.001; localisation error log-transformed: regression coefficient 0.75 [0.65, 0.85]deg, *p* < 0.001). In addition, a longer delay interval (1- vs 4-s) was also associated with worse localisation (regression coefficient 0.22[0.15, 0.30] deg of error, *p* < 0.001) but not identification (OR 0.82 [0.63, 1.07], *p* = 0.140) performance.

**Figure 2:**
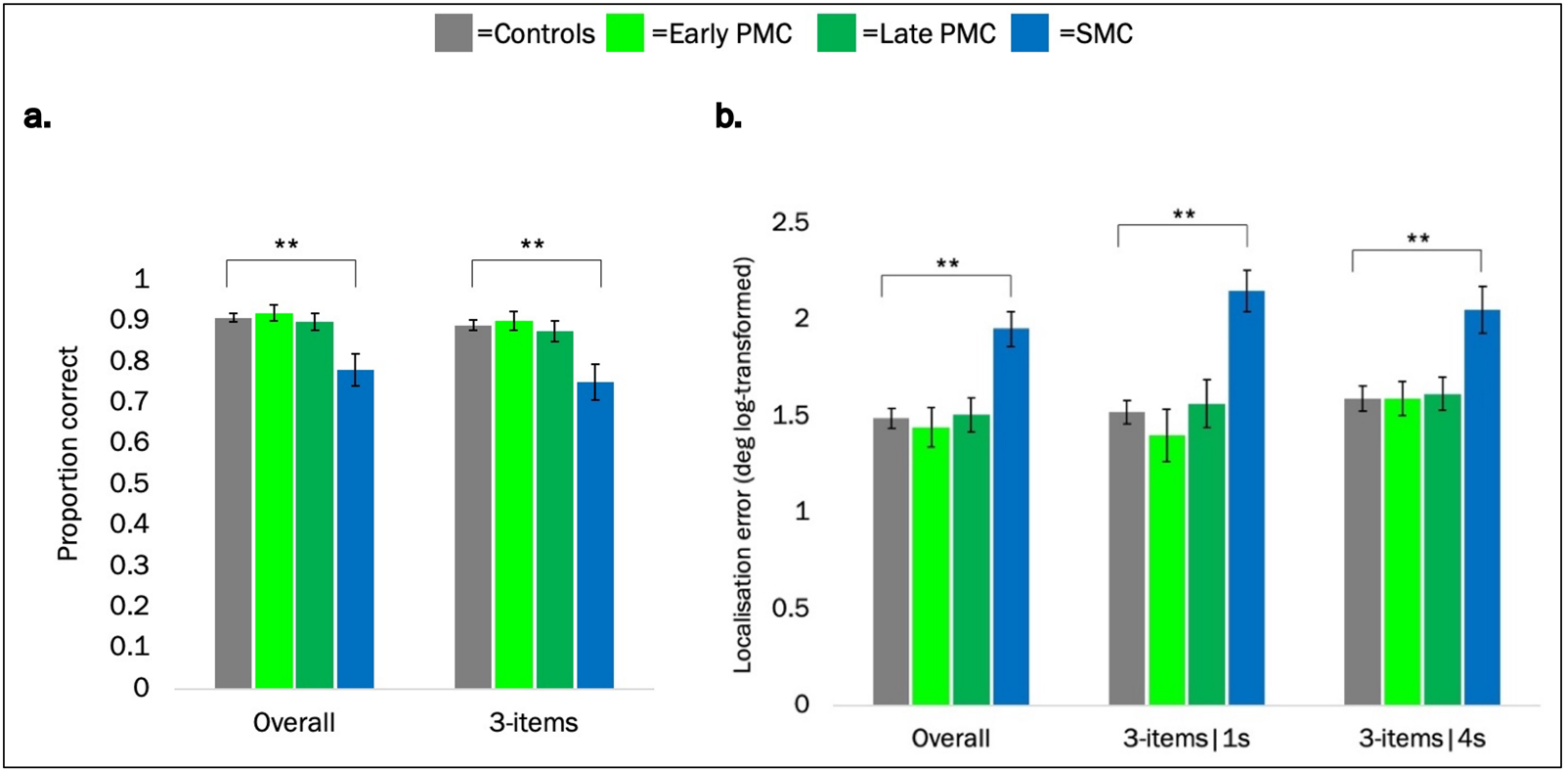
Behavioural VSTM performance by group. Adjusted mean performance (for age, sex and NART) by group. (**a**) Identification accuracy overall and for the higher memory load. (**b**) Localisation error overall and by delay for the higher memory load (deg log-transformed). Error bars represent standard errors of the group means. *PMC* presymptomatic mutation carrier, *SMC* symptomatic mutation carrier. **Significant at *p* < 0.01.

Overall, SMCs had on average 65.4 [41.5, 79.5] % lower odds of correct identification than controls (identification accuracy: adjusted mean % correct identification SMCs [95% CI] 78.1 [70.4, 85.9] % vs controls: 90.8 [88.8, 92.8] %, OR 0.35, *p* < 0.001) and significantly higher localisation error (adjusted mean log-transformed: 1.77 [5.77, 2.13] deg vs 1.49 [1.38, 1.59] deg, *p* < 0.001). No significant differences emerged in the PMC groups (early or late PMC) vs controls (identification: early: *p* = 0.618; late: *p* = 0.635; localisation: early: *p* = 0.702; late: *p* = 0.853, Fig. 2).

In the 3-item condition—the focus of eye-tracking investigations—SMCs had on average 63.1 [38.3, 78.0] % lower odds of correct identification (*p* < 0.001) and significantly higher localisation error overall (*p* < 0001) in comparison to controls. In both delay conditions localisation error (log-transformed) was greater for SMCs than controls (1-s delay: 0.91 [0.78, 1.04] deg vs 0.76 [0.53, 0.99] deg, *p* < 0.001; 4-s delay: 1.32 [1.04, 1.59] deg vs 1.06 [0.93, 1.18] deg, *p* = 0.001). No significant differences emerged in the PMC groups when compared to controls (1-s delay: early: *p* = 0.870; late: *p* = 0.205, 4-s delay: early: *p* = 0.777; late: *p* = 0.936, Fig. 2) (see Table 3 for effect size).

### Visual exploration strategies

**Figure 3:**
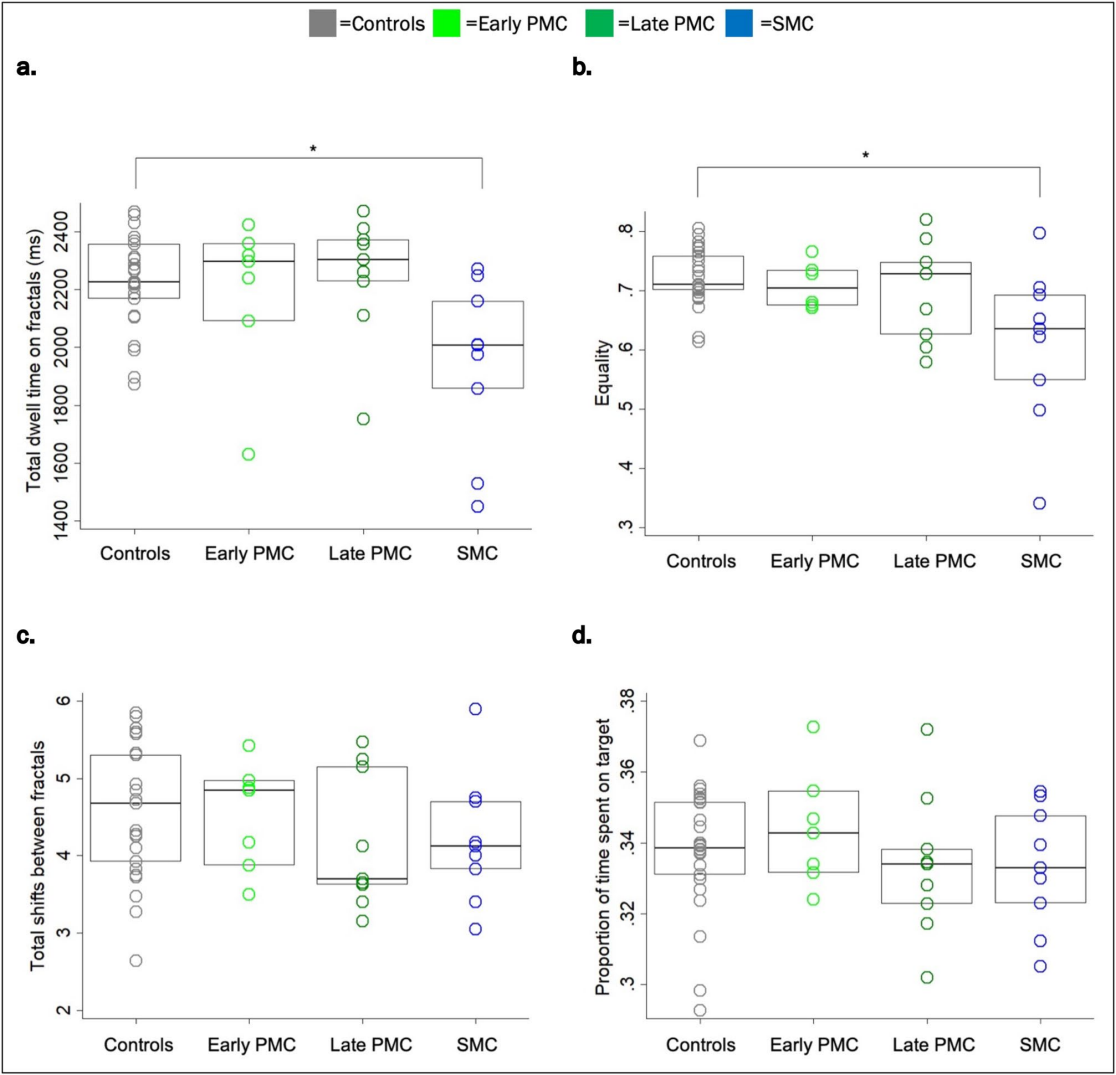
Visual exploration metrics by group. Unadjusted mean values. Each data point represents one participant, box represents median and interquartile range (IQR). (**a**) Total dwell time on fractals. (**b**) Equality index. Note that the x-axis does not start with zero. (**c**) Total shifts between fractals. (**d**) Proportion of time spent on the target fractal (unknown to the participant). *PMC* presymptomatic mutation carrier, *SMC* symptomatic mutation carrier. *Significant at *p* < 0.05.

On each trial eye-tracking metrics were compared between groups using multivariable linear regression models or bootstrapping for skewed outcomes. Results per trials were clustered by individual to account for repeated measures (see “Methods” section for details). Compared to controls, SMCs spent on average 276 ms less time fixating the stimuli (total dwell time on fractals: ‘DT’, Table 2, Fig. 3a) and showed a less homogenous distribution of fixation time among fractals (with 0.12 points lower in the equality score: ‘Eq’, Fig. 3b and a trend for fewer shifts between fractals, ‘S’, *p* = 0.181, Fig. 3c, Table 2). There was no difference between either of the PMC groups and controls (Table 2). As the target (the fractal that would be probed, ‘Pr’) was unknown to the participant at the time of viewing, there was no difference between each patient group and controls in the proportion of time spent looking at the target (Table 2, Fig. 3d).

**Table 2:** Eye-tracking metrics by group.

Adjusted mean [95% CI] Group difference [95% CI] (reference controls)				
	Controls	Early PMCs	Late PMCs	SMCs
**Visual exploration strategies**				
Total dwell time on fractals (ms)-‘DT’^a^	2224.2 [2151.0, 2297.5] NA	2197.4 [1998.5, 2396.2] − 26.9 [− 236.3, 182.5]	2258.7 [2117.3, 2400.1] 34.5 [− 126.1, 195.0]	1947.7 [1756.7, 2138.7] − **276.5 [**− **483.7, **− 69.4] *
Equality score-‘Eq’^a^	0.72 [0.70, 0.75] NA	0.71 [0.68, 0.75] − 0.01 [− 0.05, 0.03]	0.70 [0.65, 0.76] − 0.02 [− 0.08, 0.04]	0.60 [0.52, 0.68] − **0.12 [**− **0.20, **− 0.04] *
Total shifts between fractals-‘S’	4.5 [4.2, 4.9] NA	4.6 [4.0, 5.1] 0.02 [− 0.6, 0.7]	4.3 [3.7, 4.8] − 0.3 [− 0.9, 0.4]	4.1 [3.5 to 4.6] − 0.5 [− 1.1, 0.2]
Proportion of time spent on target-‘Pr'	0.34 [0.33, 0.34] NA	0.34 [0.33, 0.35] 0.004 [− 0.01, 0.02]	0.33 [0.32, 0.35] − 0.004 [− 0.02, 0.01]	0.34 [0.32, 0.35] − 0.003 [− 0.02, 0.01]
**Basic oculomotor tasks**				
Saccade amplitude (deg)^a^	4.41 [4.39, 4.44] NA	4.42 [4.36, 4.47] 0.008 [− 0.05, 0.07]	4.43 [4.39, 4.48] 0.02 [− 0.03, 0.07]	4.37 [4.31, 4.44] − 0.04 [− 0.10, 0.03]
Saccade duration (ms)^a^	39.00 [38.83, 39.16] NA	38.83 [38.51, 39.14] − 0.17 [− 0.54, 0.20]	38.98 [38.72, 39.21] − 0.03 [− 0.30, 0.25]	38.72 [38.38, 39.06] − 0.27 [− 0.63, 0.09]
Saccade velocity (deg/ms)^a^	94.84 [94.35, 95.34] NA	95.56 [94.73, 96.40] 0.72 [− 0.22, 1.65]	95.67 [94.83, 96.51] 0.83 [− 0.15, 1.80]	93.81 [92.87, 94.75] − 1.03 [2.13, 0.07]
Peak velocity (deg/ms)^a^	157.80 [152.64, 162.96] NA	156.80 [150.33, 163.27] − 1.00 [− 8.78, 6.78]	155.12 [150.49, 159.75] − 2.69 [− 9.51, 4.14]	164.20 [155.81, 172.60] 6.40 [− 3.78, 16.58]
Number of saccades per second (sacc/s)^a^	5.0 [4.9, 5.2] NA	5.1 [4.8, 5.4] 0.03 [− 0.3, 0.4]	5.0 [4.7, 5.3] − 0.08 [− 0.4, 0.3]	5.0 [4.8, 5.2] − 0.06 [− 0.3, 0.2]
Blinks per trial	5.9 [5.4, 6.4] NA	5.4 [3.7, 7.1] − 0.5 [− 2.3, 1.2]	5.6 [5.0, 6.1] − 0.4 [− 1.1, 0.4]	5.9 [5.2, 6.6] − 0.003 [− 0.9, 0.9]

*PMC* presymptomatic mutation carrier, *SMC* symptomatic mutation carrier, *NA* not applicable. Bold = significant; *significant at *p* < 0.05

^a^From bias-corrected and accelerated (BCA) approach.

Compared to controls, basic oculomotor characteristics revealed no significant differences in any of the patient groups (Table 2) and while the blinks were removed from analysis, a separate investigation revealed a similar number between groups (Table 2).

### Visual exploration strategies as predictors of VSTM performance

Multivariable regression models revealed that across the whole sample, increasing dwell time on fractals and a higher equality score were independently associated with greater odds of correct identification and smaller localisation error. Across all groups, for every 100 ms increase in the total dwell time on fractals for a given trial, the odds of correct identification increased by 4.06 [0.80, 7.33] % (*p* = 0.015) and resulted in a 2.07 [0.64, 3.50] % decrease of localisation error (*p* = 0.006) in that trial. Similarly, a higher equality score, ‘Eq’, resulted in greater odds of correct identification (*p* = 0.006) and a reduction of localisation error (*p* = 0.021). To put this into context, with Eq = 0.5 *vs* Eq = 1, in a trial, the percentage of correct identification was 83.21 [80.16, 86.25] % vs 89.50 [86.55, 92.46] % and localisation error (deg, log-transformed) was 1.79 [1.70, 1.88] deg vs 1.64 [1.51, 1.76] deg, respectively. Both of these effects remained when excluding SMCs (identification and DT: *p* = 0.025; identification and Eq: *p* < 0.001; localisation and DT: *p* = 0.035, localisation and Eq: *p* = 0.021).

Neither identification (*p* = 0.291) nor localisation error (*p* = 0.266) were significantly associated with the number of eye movement shifts (saccades) between fractals.

Increasing time spent looking at the target (in proportion to the overall time spent on all fractals), ‘Pr’, was significantly associated with greater odds of correct identification*/*decreasing error (OR 2.82, *p* = 0.037). To put this into context, Pr = 0.33 (33% of fixations spent on the target) = 86.0 [83.7, 88.25] % correct identification vs Pr = 1: 92.26 [87.22, 97.31] %. A weak trend in the same direction was observed between the time spent looking at the target and localisation error although this did not reach statistical significance (*p* = 0.128).

**Figure 4:**
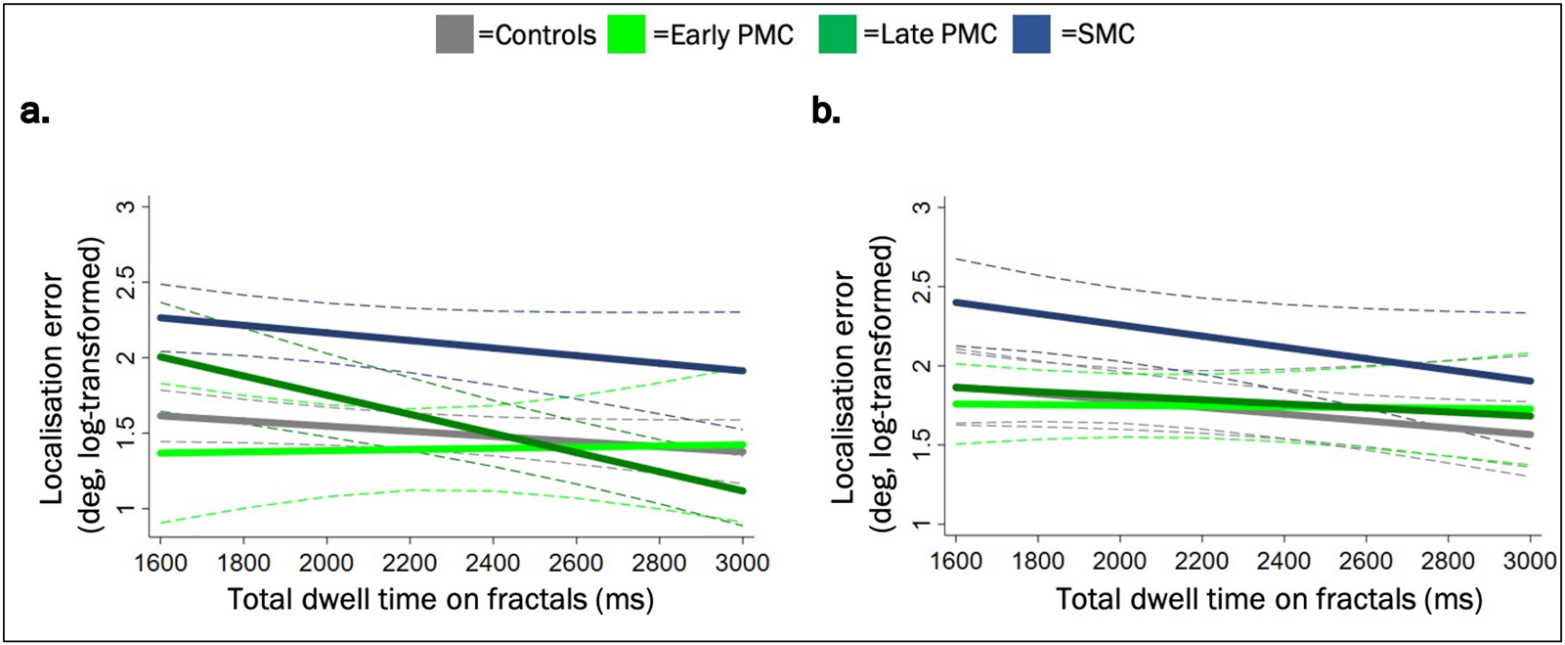
(**a**) 1-s delay: localisation error by the total dwell time on fractals (DT). (**b**) 4-s delay: localisation error by the total dwell time on fractals (DT). Lines represent best fit lines of the DT × group interaction from the multivariable regression model for each delay condition with localisation error as the outcome and NART, sex and DT as predictors. The dotted lines represent 95% confidence intervals. *PMC* presymptomatic mutation carrier, *SMC* symptomatic mutation carrier.

For localisation error, there was a significant interaction between group, delay and the total dwell time on fractals, whereby for every 100 ms increase in the total dwell time on fractals in a trial, late PMCs showed a *smaller* localisation error in the 1-s vs 4-s delay condition compared to controls (interaction coefficient = − 5.73 [− 10.47, − 1.00] %, *p* = 0.019). This suggested a *stronger association* between fixation time and error for late PMCs compared to controls, specific to the 1-s delay condition and not observed in the other patient groups (early PMCs: − 0.15 [− 5.47, 5.16] %, *p* = 0.954; SMCs: 0.32 [− 5.08, 5.71] %, *p* = 0.907). More specifically, in the 1-s delay condition, for every 100 ms increase in the total dwell time on fractals, localisation error decreased by 4.64 [1.00, 8.28] % more in late PMCs compared to controls (*p* = 0.014). Taken together, these findings suggested that with a rather short fixation time on fractals, localisation error was greater for late PMCs than for controls (Fig. 4a). No significant interactions between group and the total dwell time on fractals emerged in other groups in this condition (early PMC: *p* = 0.498, SMCs: *p* = 0.672), nor in the 4-s delay condition (early PMC: *p* = 0.326, late PMC: *p* = 0.675 and SMCs: *p* = 0.508, Fig. 4b) or between group, delay and the equality score (early PMC: *p* = 0.628, late PMC: *p* = 0.388; SMCs: *p* = 0.355).

For identification performance, there were no significant interactions (DT: early PMC: *p* = 0.070; late PMC: *p* = 0.906; SMCs: *p* = 0.162; Eq: early PMC: *p* = 0.982, late PMC: *p* = 0.801; SMCs: *p* = 0.262 and Pr: early PMC: *p* = 0.291, late PMC: *p* = 0.172; SMCs: *p* = 0.159). As there was no overall effect of delay on identification accuracy, interaction tests with delay were not pursued.

We next examined the effect of including visual exploration strategies (DT, Eq, S and Pr) as additional predictors in regression models of VSTM performance. All effect sizes remained small and non-significant but there was some indication that including these predictors resulted in a slight *increase* between late PMCs and controls and *decrease* between SMCs and controls in localisation performance, mainly in the 1-s condition (Table 3).

**Table 3:** Visual exploration strategies as predictors of VSTM performance.

Delay	Adjusted mean [95% CI] Group difference [95% CI] (control as reference)				
	Model	Controls	Early PMCs	Late PMCs	SMCs
**Identification accuracy: % correct and Odds Ratio for correct response**					
Across delays	Adjusted by NART, sex and delay^a^	88.8 [86.1, 91.4] NA	88.8 [84.0, 93.7] 1.01 [0.57, 1.77]	87.6 [82.5, 92.7] 0.89 [0.52, 1.54]	75.1 [66.7, 83.8] 0.38 [0.22, 0.64]**
	Additionally, adjusted by: DT, Eq S and Pr	88.4 [85.9, 90.9] NA	88.2 [83.7, 92.8] 0.99 [0.59, 1.66]	87.2 [82.3, 92.0] 0.89 [0.54, 1.48]	77.7 [70.0, 85.4] 0.45 [0.26, 0.76]**
**Localisation performance: error in deg (log-transformed) and % difference**					
Across delays	Adjusted by NART, sex and delay^a^	1.62 [1.49, 1.74] NA	1.64 [1.47, 1.82] 2.37 [− 17.61, 27.19]	1.67 [1.45, 1.89] 5.26 [− 18.16, 35.39]	2.23 [2.02, 4.43] 84.11 [44.72, 134.21]**
	Additionally, adjusted by: DT, Eq S and Pr	1.62 [1.51, 1.73] NA	1.65 [1.51, 1.79] 3.08 [− 14.20, 23.83]	1.69 [1.48, 1.91] 7.84 [− 15.16, 37.08]	2.18 [2.00, 2.36] 75.15 [41.38, 116.97]**
1-s	Adjusted by NART, sex and delay^a^	1.51 [1.38, 1.63] NA	1.49 [1.26, 1.72] − 1.68 [− 23.42, 26.23]	1.56 [1.30, 1.82] 5.72 [− 19.75, 39.27]	2.17 [1.95, 2.38] 93.62 [50.48, 149.14]**
	Additionally, adjusted by: DT, Eq S and Pr	1.51 [1.39, 1.62] NA	1.50 [1.29, 1.71] − 0.62 [21.44, 25.73]	1.59 [1.34, 1.84] 8.60 [− 17.46, 42.89]	2.11 [1.92, 2.31] 39.31 [44.54, 132.89]**
4-s	Adjusted by NART, sex and delay^a^	1.73 [1.56, 1.90] NA	1.79 [1.60, 1.99] 6.21 [− 17.49, 36.73]	1.78 [1.78, 1.98] 4.83 [− 17.76, 33.63]	2.29 [2.03, 2.55] 74.67 [29.59, 135.44]**
	Additionally, adjusted by: DT, Eq S and Pr	1.74 [1.59, 1.89] NA	1.79 [1.60, 1.98] 5.69 [− 16.90, 34.42]	1.80 [1.61, 2.00] 6.90 [− 15.28, 34.89]	2.24 [2.00 to 2.49] 65.90 [24.38, 121.26]**

*PMC* presymptomatic mutation carrier, *SMC* symptomatic mutation carrier, *CI* confidence intervals, *DT* total dwell time on fractals, *Eq* Equality, *S* total number of shifts between fractals, *Pr* proportion of time spent looking at the target, *NA* not applicable.

^a^In order to allow for comparison between models, participants with a score > 2.5 SD in a visual exploration strategy metric (n = 2) were excluded. Of note, as delay did not have a significant effect on identification accuracy investigations were not pursued by delay for this metric. Bold = significant; **significant at *p* < 0.01.

## Discussion

In this study we investigated eye movements as predictors of VSTM function in FAD. Memory performance for the target identity and target location were measured using a delayed-reproduction paradigm^21^ in a continuous analogue scale which quantified the *precision* of memory recall. The key finding was that the relationship between eye movements during encoding—indexed indirectly by the overall time spent fixating the stimulus—and VSTM performance differed between presymptomatic FAD mutation carriers and controls.

Across the sample as a whole, several measures of visual exploration strategies predicted behavioural task performance. The time spent fixating the stimuli (total dwell time) in a trial and a more equal distribution of this time among the three items were both associated with better recall of object identity and location. As expected, the proportion of time spent fixating the target fractal (the item that was later probed) was also associated with better identification performance and trend in the same direction observed for localisation error. The total number of saccadic shifts was not associated with recall for the target identity or the target location.

Compared to controls, SMCs showed a shorter dwell time on fractals and a less homogenous distribution of this fixation time among the three fractals (with a lower equality score and a trend for fewer shifts between fractals). While these findings may be the result of a slower exploration strategy, a lack of engagement with the stimuli and fixations on other parts of the screen, may also be a valid explanation. Low-level eye movement deficits have been reported in sporadic AD, including impaired anti-saccade performance^28^ and longer saccade latency^29^. Yet, differences in oculomotor characteristics were not observed between FAD (presymptomatic or symptomatic) mutation carriers and controls. This raises important considerations with regards to different oculomotor characteristics between in sporadic and genetic forms AD and should be explored in future studies.

Consistent with the literature that memory for object identity and location improves with the number and duration of fixations in a cumulative manner^22^ and with increasing exploration of different aspects of an object or a scene (the ‘exploration–exploitation dilemma’), we propose that the viewing behaviour observed in SMCs may be at the root of some of the resulting behavioural VSTM impairments. More specifically, if the time spent viewing the stimuli was neither ‘optimal’ (evidenced by the high inequality in fixation distribution among all three fractals) nor ‘sufficient’ (evidenced by the lower dwell time), encoding may be viewed as ‘ineffective’—particularly as these eye movements were associated with poorer task performance. In line with this, the group difference between SMCs and controls was smaller after adjustment for visual exploration strategy metrics.

Compared to controls, late PMCs showed a stronger reliance on the total stimuli fixation time for accurate localisation performance in the 1-s delay condition. Yet, if accurate performance relies on fixation time (as a proxy to encoding time^15^), why might the relationship between dwell time and localisation error be stronger in late PMC individuals compared to controls? Our findings show that within shorter dwell times (1800–2200 ms), late PMCs had a less accurate localisation performance than controls, but following a longer dwell time, performance increased considerably. The fixation time required to achieve an accurate encoding was therefore longer for late PMCs than controls, suggesting it took late PMCs longer to effectively encode the stimuli. More broadly, this indicates that the integrity and efficiency of encoding processes might be weakening in late PMCs due to the advancing preclinical AD state. This effect was not observed in early PMCs who, further away from expected onset, did not present with a similar pattern. Additionally, the narrowing window between the time required to encode, and the time available to encode during a fixed presentation time, may have led to a reduction in the variability of dwell times associated with subsequent accurate localisation performance (i.e. stronger association for late PMCs than controls). The hypothesis presented here is comparable to that suggested by Bondi and colleagues in episodic memory, whereby another group of individuals at-risk of AD (by virtue of the *APOE* ε4 allele) required additional cognitive effort to achieve comparable performance levels on tests of episodic memory encoding^30^. Accounting for visual exploration strategy metrics yield somewhat higher localisation error for late PMCs compared to controls: with the same total dwell time on fractals, late PMCs had worse localisation error than controls. Yet, this was the case for short (~ 1800 ms) fixation times and the effect size remained small, suggesting this weakening effect may be too subtle to be reflected in VSTM task outcomes. Therefore, although somewhat speculative, these findings indicate that with a shorter fixation time, larger differences in localisation error would be observed between late PMCs and controls and this hypothesis merits further investigation.

Localisation error is a measure of the distance from the exact location of the *target* to the position selected by the participant (for correctly identified objects). As the participant chooses the fractal and *then* places it in the remembered location, from a theoretic point of view, localisation performance may thus represent a measure of ‘*correct binding’* in a *continuous scale* (of the object’s identity to its correct location). So, why was the stronger association between dwell time and localisation error in the late PMC group only seen in the 1-s delay condition? As reported previously^3,21^, longer 4-s delays lead to poorer performance across all subjects. Pertzov and colleagues argue this may relate to the erosion of the representation in memory due to the limitations of the episodic memory buffer (the time over which the object’s representations are maintained in memory)^21,22^. As memory of the object’s identity and location are thought to be held in different brain regions^21,31–33^ and not tightly bound in the episodic buffer, they need to be actively linked *over time* for the correct recall of which object was where. Such effects may therefore mask the more subtle relationship between dwell time and localisation error—which is more reflective of processes at encoding than processes during maintenance and retrieval. Notably, this result may also be explained by the attention and frontal/executive demands of this task (with the localisation measure being particularly sensitive due to its continuous nature), rather than the visuospatial or memory aspects per se—although PMCs did not show evidence of such cognitive deficits in more traditional neuropsychology tasks.

The current study has a number of limitations. First, the sample size was relatively small due to the rarity of FAD and the limited number of symptomatic individuals who are able to perform the task. As a result, our sample included mutation carriers from pedigrees with different *PSEN1* and *APP* mutations and some of the variance observed within groups may reflect underlying genetic differences. However, creating subgroups by mutation type would not have been possible also due to issues around validity of modelling such small groups. Second, normal ageing has been associated with lower VSTM performance in this task^27^ raising concerns about the specificity of such impairments^3^. Nevertheless, as previously argued by Liang et al.^8^, the highest predictive power lies in the comparison of individuals who will develop AD to *age-matched controls* who do not share the same risk factors. From an eye-tracking perspective, age-related differences in viewing patterns have been described in the literature^34,35^ but the extent to which these contribute to memory and hippocampal activity in older adults is still unknown^36^. Crucially, our most significant finding was in the late PMC group that is well-matched for age. Third, to our surprise, SMCs scored significantly lower than controls in the traditional object perception task (visual and object space perception test—VOSP) possibly raising concerns for the contribution of perceptual impairments to our findings. Previous reports using this task have argued that the detrimental effect of increasing memory load and retention duration on VSTM performance, are strong indicators of a memory rather than perceptual impairment. While we evaluated lower-level oculomotor characteristics, we did not test visual acuity and this remains a limitation of the design. Importantly, investigations of perceptual impairments, for example involving visual impairments, are lacking in presymptomatic FAD. In view of some preclinical (e.g. Ref.^37^) and symptomatic reports (e.g. Ref.^38^) in sporadic AD, perceptual deficits should be explored in future FAD research. Lastly and similar to previous reports from our centre^3^, late PMCs had lower education levels compared to controls and while VSTM tasks like the one presented here, have been suggested as impervious to education and intercultural background^39,40^, this requires further exploration.

In conclusion, we present the first characterization of viewing behaviour of a group of symptomatic and presymptomatic FAD mutation carriers performing a VSTM task. Our exploratory findings provide further insight into the nature of the early memory impairments in this population and support the hypothesis of a ‘weakened encoding’ process in PMCs within 6 years to expected symptom onset. Importantly, future research using other analysis and techniques such as time course analysis and functional magnetic resonance imaging, may help increase our understanding of impairments in memory processes like encoding deficits and evaluate the extent to which these are specific to the association or binding of features in working memory or to encoding of individual features per se. Consistent with the notion that VSTM tasks may be sensitive cognitive markers of preclinical AD, we provide novel ways to exploit its potential.

## Methods

### Participants and study design

Participants were recruited from an ongoing longitudinal FAD study at the Dementia Research Centre, University College London (UCL), which receives referrals from across the UK. Participants were recruited into the study if there was an autosomal dominant family history of AD and a known pathological mutation in *PSEN1* or *APP* genes in at least one affected family member. Healthy individuals (without a family history of AD) were also recruited to the study from our research database.

Mutation analysis was carried out using Sanger sequencing^41,42^. Individuals with novel variants in *PSEN1* or *APP* were assessed for the presence of additional mutations in other dementia-related genes using the Medical Research Council Dementia Gene Panel^43^. Genetic results were available for all at-risk individuals, on either a clinical or a research basis. Research genetic results were only shared with the statistician involved in the study and not disclosed to the participants or to other researchers who remained blind to whether presymptomatic individuals were mutation carriers or non-carriers.

As per Liang and colleagues, we used estimated years to/from symptom onset (EYO) as an approximation of how far individuals (presymptomatic and symptomatic) were from symptom onset^3^. EYO was calculated for the mutation carriers by subtracting the age at which their parent first developed progressive cognitive symptoms from the participant’s age^44^. We considered EYO in our group classification to account for differences which might affect preclinical cognitive changes at the time of assessment. Individuals were thus classified as: SMC, ‘early’ PMC (more than 6 years from EYO), ‘late’ PMC (within 6 years from EYO) or control. Symptomatic individuals were those who had a positive genetic test and cognitive symptoms consistent with AD and scored higher than zero on the Clinical Dementia Rating Scale (CDR)^45^. PMCs were at-risk individuals who had a positive genetic test but did not have symptoms and who scored zero on the CDR scale^45^. Control participants consisted of both non-carriers (at-risk individuals who tested negative for pathological mutations) and healthy individuals (from our research database) recruited for the study. The cut-off of 6 years corresponded to the median split of PMCs in our dataset.

All participants underwent clinical assessment, a semi-structured interview, neurological examination and the CDR scale^45^, subjective cognitive decline questionnaires (MyCog^46^, AD8^47^); depression and anxiety questionnaires (HADS)^48^ and completed a standard neuropsychology battery. The battery measured several cognitive domains, including: episodic memory (recognition memory test—RMT for words and faces^49^); working memory (digit span^50^); intellectual function (Wechsler abbreviated scale of intelligence—WASI^51^); executive function (Stroop^52^); confrontational naming (graded naming test—GNT^53^); vocabulary (British picture vocabulary scale—BPVS^54^); arithmetic (graded difficulty arithmetic test—GDA^55^); visual perception (visual object and apace perception battery: object decision—VOSP OD^56^); processing speed (digit symbol test^57^) and estimated premorbid intelligence (national adult reading test—NART^58,59^ (Table 1).

All methods were performed in accordance with the relevant guidelines and regulations. The study was approved by The National Hospital for Neurology and Neurosurgery and Institute of Neurology Joint Research Ethics Committee (subsequently, National Research Ethics Service Committee, London Queen Square, REC ref 11/LO/0753). Written informed consent was obtained from all participants.

### Object-localisation VSTM task

The study protocol included the *“*Object-localisation” VSTM task^21^, run on a Dell 2120 desktop computer with a 23-inch screen with a 1920 × 1080-pixel matrix corresponding to approximately 62 × 35° of visual angle at a viewing distance of 42 cm. All objects including the foils were drawn from a pool of 60 fractals that were used across the experiment (rendered using http://sprott.physics.wisc.edu/fractals.htm). Following Liang and colleagues’ finding that testing confined to only 50 trials was sufficient to distinguish FAD cases from controls^3^; the experiment consisted of 50 trails. Saccades were defined using the standard velocity and acceleration thresholds recommended by Eyelink (30°/s and 8000°/s^2^). Periods between saccades were defined as fixations. A 9-point calibration and validation were performed prior to the experiment. All the data were obtained from recordings with an average Cartesian prediction error of < 1° during the validation. A drift correction procedure was used before each individual trial.

In addition to identification accuracy and localisation error, two additional outcome metrics of task performance have been used in previous publications^3,21^: swap errors (the percentage of correctly identified objects placed within 4.5° eccentricity of other fractals in the original array) and nearest item control (the distance between the centre of the target object once placed in its remembered location and the location of the nearest fractal from the memory array). Results for these metrics are reported in the Supplementary Materials (e.g. Supplementary Fig. S1).

### Eye-tracking metrics

All eye-tracking recordings were visually inspected using Data Viewer to check for any signal loss that would interfere with data analysis and interpretation of results. Blinks were identified and removed using Eyelink’s automated blink detection. Vision was binocular but only eye movements from the right eye were recorded.

In order to test the hypothesis that encoding—indexed indirectly by the overall time spent fixating a stimulus—might be particularly affected in presymptomatic FAD individuals, we examined four metrics related to perception of the stimuli. An assumption that we wanted to check was whether one reason for variable or poor performance was not having spent an equal amount of time processing the details of each fractal. This metric will be referred here as “equality” (see below).

The eye-tracking measures capturing exploration strategies (for each trial) during the 3-s viewing period were:

- Total dwell time on fractals: sum of the total fixation time on all fractals.
- Proportional time spent looking the target: time spent fixating the target (the item that was later probed) divided by the total time spent fixating all three-items.
- Equality: homogeneity in the distribution of the time spent fixating on the three-items. We generated a metric between 1 and 0, where 1 represents a completely equal distribution of fixation time between the three fractals (f) i.e. f1 = 1000 ms; f2 = 1000 ms; f3 = 1000 ms and anything lower than 1 represents a less homogenous or less equal distribution of fixation time e.g. f1 = 3000 ms; f2 = 0 ms; f3 = 0 ms or f1 = 1800 ms; f2 = 1200; f3 = 0 ms.
- Total number of shifts between stimuli: total number of eye movements between the three items.

**Figure 5:**
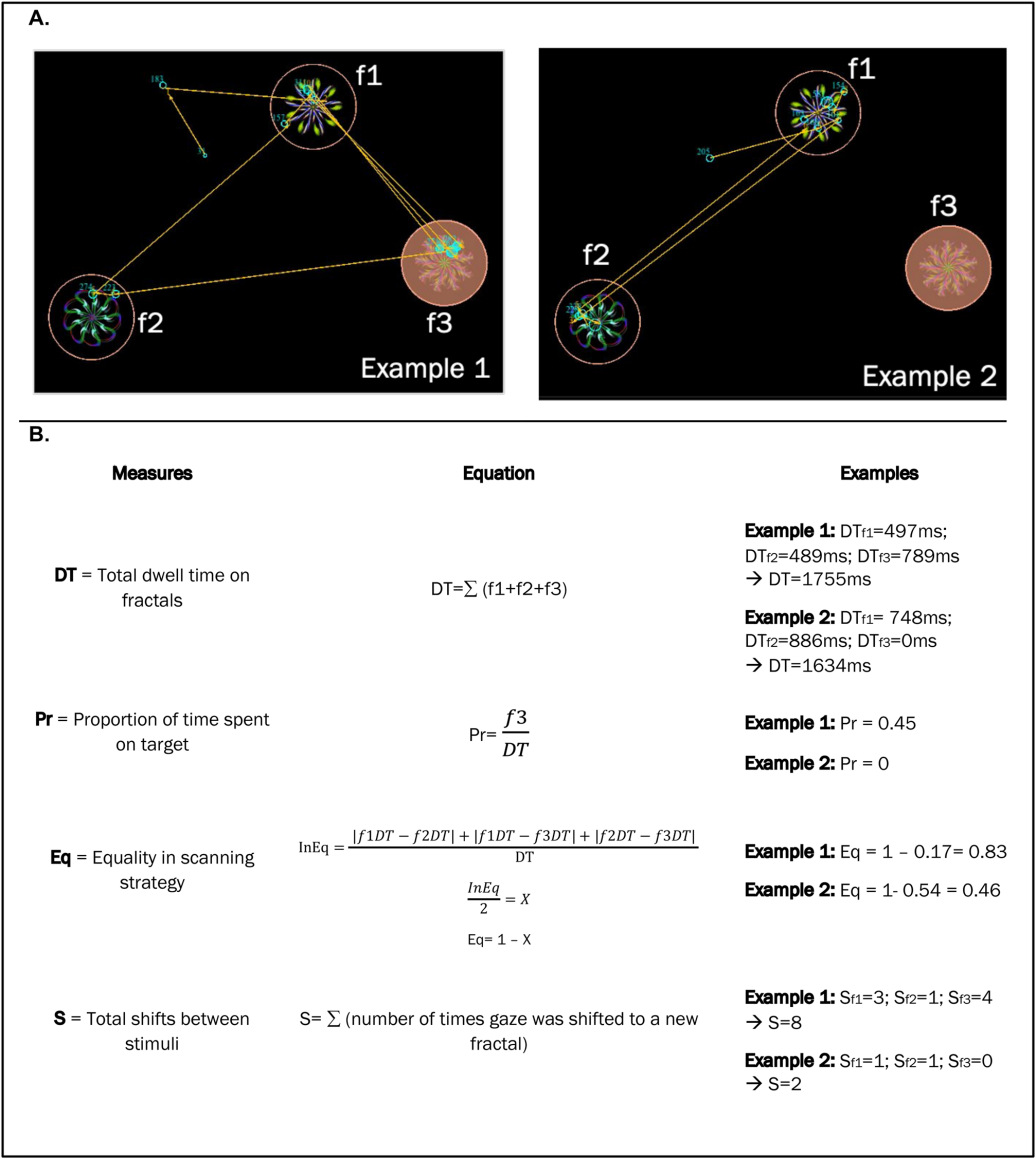
(**a**) Example of the stimuli. Highlighted fractal represents the target (f3), the identity of which was not known to the participant at the time of viewing. Cyan circles show fixations, yellow arrows indicate saccades and the circles around each fractal outline the region of interest. (**b**) Definition of visual exploration measures with examples from sample array. InEq = Inequality. f1, f2 and f3 are fractal 1, 2, and 3 respectively. DT_f1_, DT_f2_ and DT_f3_ are the dwell times on fractal 1, 2, and 3 respectively. S_f1_, S_f2_ and S_f3_ are the total shifts on fractal 1, 2 and 3 respectively.

For examples of the stimuli and formulas used to generate each metric see Fig. 5.

In order to account for any low-level oculomotor differences, we also evaluated basic oculomotor metrics (defined for each trial) between groups using Eyelink’s automated detection algorithm:

- Saccade amplitude (deg): average amplitude of each saccade.
- Saccade velocity (deg/ms): average velocity of each saccade.
- Peak saccade velocity (deg/ms): the highest velocity reached during the saccade.
- Saccade duration (ms): average time between the start of a saccade and its end.
- Number of saccades per second (saccades/s): The number of saccades that were made after the target appeared, excluding blinks (disappearance of the pupil) and excluding saccades smaller than 2 degrees^60^.

### Statistical analysis

Baseline demographics and neuropsychology scores were compared between controls and each of SMCs, late PMCs and early PMCs using ANOVA (age; NART; verbal and performance IQ; arithmetic; digit span backwards; verbal and category fluency; GNT; spatial digit span forwards or backwards) or Kruskal–Wallis test where the distribution of the variable was skewed (MMSE; education level-in years; CDR score; anxiety; depression; self-reports of subjective decline, measured via the MyCog and AD8 scales; RMT for faces and words; digit span forwards; BPVS; VOSP OD; Stroop; Camden PAL; digit symbol; Trails). Normality was tested using the Shapiro–Wilk normality test. After this, either parametric or non-parametric (Dunn’s test) post-hoc pairwise comparisons were used to investigated where differences were observed. Fishers’ exact test was used to compare the sex distribution between the groups instead of Chi-squared test as this is more appropriate for smaller sample sizes.

Behavioural performance on the VSTM task was compared between controls and patient groups. Due to a skewed distribution the absolute localisation error for each trial was log-transformed and analysed using a linear regression model. Analysis of object identity used a logistic regression model. Models used robust standard errors to account for clustering by participant. Regression analysis was used in order to allow analysis of the full trial-by-trial data which was available.

Visual exploration metrics and basic oculomotor characteristics, on each trial, were compared between controls and patient groups using multivariable linear regression models. Examination of residuals was performed to check model fits. For outcomes with skewed distributions (saccade amplitude, saccade duration, average saccade velocity, peak velocity, DT and Eq) bootstrapping, clustered on individual to account for repeated measures, was used to produce bias-corrected and accelerated (BCA) 95% confidence intervals (CIs) from 2000 replications.

To investigate the relationship between VSTM function and viewing behaviour we used multivariable linear or logistic regression models, where the outcome was the VSTM metric on each trial (identification accuracy or log of localisation error) and the predictors were group, sex, age at assessment, NART scores and the visual exploration strategy metrics (DT, Pr, Eq and S). Interactions were examined between visual exploration strategy metrics and group and between visual exploration strategy metrics, group and delay in relation to VSTM performance where relevant.

All models were adjusted for sex, age, NART and delay (1- vs 4-s). As saccade amplitude, velocity and duration are closely linked to one another, they were each included as covariates in corresponding models.

For each variable, participants were excluded if their overall performance deviated by 2.5 standard deviations (SD) from either side of the mean of each group (a total of n = 3 were excluded: n = 1 due to loss of eye-tracking signal throughout the experiment; n = 1 due to the DT score and n = 1 due to the Eq score). Statistical significance threshold was set to *p* < 0.05 and all analysis performed on Stata v.14.

## Supplementary Information

Supplementary Information.

## Data Availability

The datasets generated during and/or analysed during the current study are available in an anonymized format (to avoid unblinding of genetic status) from the corresponding author on reasonable request.

## References

[1] Parra MA, Abrahams S, Logie RH, Sala SD (2009). Age and binding within-dimension features in visual short-term memory. Neurosci. Lett..

[2] Ma WJ, Husain M, Bays PM (2014). Changing concepts of working memory. Nat. Neurosci..

[3] Liang Y, Pertzov Y, Nicholas JM, Henley SMD, Crutch S, Woodward F (2016). Visual short-term memory binding deficit in familial Alzheimer’s disease. Cortex.

[4] Parra MA, Abrahams S, Logie RH, Méndez LG, Lopera F, Della SS (2010). Visual short-term memory binding deficits in familial Alzheimer’s disease. Brain J. Neurol..

[5] Pavisic IM, Suarez-Gonzalez A, Pertzov Y (2020). Translating visual short-term memory binding tasks to clinical practice: From theory to practice. Front. Neurol..

[6] Didic M, Barbeau EJ, Felician O, Tramoni E, Guedj E, Poncet M (2011). Which memory system is impaired first in Alzheimer’s disease?. J. Alzheimers Dis. JAD..

[7] Parra MA (2017). A commentary on Liang et al.’s paper with regard to emerging views of memory assessment in Alzheimer’s disease. Cortex.

[8] Liang Y, Pertzov Y, Henley S, Woodward F, Husain M, Crutch S (2017). Visual short-term memory binding deficits in Alzheimer’s disease: A reply to Parra’s commentary. Cortex.

[9] Bays PM, Husain M (2008). Dynamic shifts of limited working memory resources in human vision. Science.

[10] Wilken P, Ma WJ (2004). A detection theory account of change detection. J. Vis..

[11] Zokaei N, Burnett Heyes S, Gorgoraptis N, Budhdeo S, Husain M (2015). Working memory recall precision is a more sensitive index than span. J. Neuropsychol..

[12] Posner MI (1980). Orienting of attention. Q. J. Exp. Psychol..

[13] Milea D, Lobel E, Lehéricy S, Pierrot-Deseilligny C, Berthoz A (2005). Cortical mechanisms of saccade generation from execution to decision. Ann. N. Y. Acad. Sci..

[14] Grady CL, Furey ML, Pietrini P, Horwitz B, Rapoport SI (2001). Altered brain functional connectivity and impaired short-term memory in Alzheimer’s disease. Brain J. Neurol..

[15] Hannula DE, Althoff RR, Warren DE, Riggs L, Cohen NJ, Ryan JD (2010). Worth a Glance: Using eye movements to investigate the cognitive neuroscience of memory. Front. Hum. Neurosci..

[16] Hollingworth A, Williams CC, Henderson JM (2001). To see and remember: Visually specific information is retained in memory from previously attended objects in natural scenes. Psychon. Bull. Rev..

[17] Fernández G, Orozco D, Agamennoni O, Schumacher M, Sañudo S, Biondi J (2018). Visual processing during short-term memory binding in mild Alzheimer’s disease. J. Alzheimers Dis. JAD..

[18] Duc AH, Bays P, Husain M (2008). Eye movements as a probe of attention. Prog. Brain Res..

[19] Kietzmann TC, König P (2015). Effects of contextual information and stimulus ambiguity on overt visual sampling behavior. Vis. Res..

[20] Tatler BW, Brockmole JR, Carpenter RHS (2017). LATEST: A model of saccadic decisions in space and time. Psychol. Rev..

[21] Pertzov Y, Dong MY, Peich M-C, Husain M (2012). Forgetting what was where: The fragility of object-location binding. PLoS ONE.

[22] Pertzov Y, Avidan G, Zohary E (2009). Accumulation of visual information across multiple fixations. J. Vis..

[23] Rayner K, Smith TJ, Malcolm GL, Henderson JM (2009). Eye movements and visual encoding during scene perception. Psychol. Sci..

[24] Weston PSJ, Poole T, Nicholas JM, Toussaint N, Simpson IJA, Modat M (2020). Measuring cortical mean diffusivity to assess early microstructural cortical change in presymptomatic familial Alzheimer’s disease. Alzheimers Res. Ther..

[25] Baddeley A (2003). Working memory: Looking back and looking forward. Nat. Rev. Neurosci..

[26] Goldstein FC, Loring DW, Thomas T, Saleh S, Hajjar I (2019). Recognition memory performance as a cognitive marker of prodromal Alzheimer’s disease. J. Alzheimers Dis..

[27] Pertzov Y, Heider M, Liang Y, Husain M (2015). Effects of healthy ageing on precision and binding of object location in visual short term memory. Psychol. Aging..

[28] Peltsch A, Hemraj A, Garcia A, Munoz DP (2014). Saccade deficits in amnestic mild cognitive impairment resemble mild Alzheimer’s disease. Eur. J. Neurosci..

[29] Yang Q, Wang T, Su N, Xiao S, Kapoula Z (2013). Specific saccade deficits in patients with Alzheimer’s disease at mild to moderate stage and in patients with amnestic mild cognitive impairment. Age Dordr Neth..

[30] Bondi MW, Houston WS, Eyler LT, Brown GG (2005). fMRI evidence of compensatory mechanisms in older adults at genetic risk for Alzheimer disease. Neurology.

[31] Darling S, Della Sala S, Logie RH, Cantagallo A (2006). Neuropsychological evidence for separating components of visuo-spatial working memory. J. Neurol..

[32] Kessels RPC, Postma A, de Haan EHF (1999). P and M channel-specific interference in the what and where pathway. NeuroReport.

[33] Postma A, Kessels RPC, van Asselen M (2008). How the brain remembers and forgets where things are: The neurocognition of object–location memory. Neurosci. Biobehav. Rev..

[34] Chan JPK, Kamino D, Binns MA, Ryan JD (2011). Can changes in eye movement scanning alter the age-related deficit in recognition memory?. Front. Psychol..

[35] Shih S-I, Meadmore KL, Liversedge SP (2012). Aging, eye movements, and object-location memory. PLoS ONE.

[36] Voss JL, Bridge DJ, Cohen NJ, Walker JA (2017). A closer look at the hippocampus and memory. Trends Cogn. Sci..

[37] Santos CY, Johnson LN, Sinoff SE, Festa EK, Heindel WC, Snyder PJ (2018). Change in retinal structural anatomy during the preclinical stage of Alzheimer’s disease. Alzheimers Dement. Amst. Neth..

[38] Yoon SP, Grewal DS, Thompson AC, Polascik BW, Dunn C, Burke JR (2019). Retinal microvascular and neurodegenerative changes in Alzheimer’s disease and mild cognitive impairment compared with control participants. Ophthalmol. Retina..

[39] Parra MA, Sala SD, Abrahams S, Logie RH, Méndez LG, Lopera F (2011). Specific deficit of colour–colour short-term memory binding in sporadic and familial Alzheimer’s disease. Neuropsychologia.

[40] Yassuda MS, Carthery-Goulart MT, Cecchini MA, Cassimiro L, Fernandes KD, Baradel RR (2019). Free recall of bound information held in short-term memory is unimpaired by age and education. Arch. Clin. Neuropsychol..

[41] Janssen JC, Beck JA, Campbell TA, Dickinson A, Fox NC, Harvey RJ (2003). Early onset familial Alzheimer’s disease: Mutation frequency in 31 families. Neurology.

[42] Ryan NS, Nicholas JM, Weston PSJ, Liang Y, Lashley T, Guerreiro R (2016). Clinical phenotype and genetic associations in autosomal dominant familial Alzheimer’s disease: A case series. Lancet Neurol..

[43] Beck J, Pittman A, Adamson G, Campbell T, Kenny J, Houlden H (2014). Validation of next-generation sequencing technologies in genetic diagnosis of dementia. Neurobiol. Aging..

[44] Ryman DC, Acosta-Baena N, Aisen PS, Bird T, Danek A, Fox NC (2014). Symptom onset in autosomal dominant Alzheimer disease. Neurology.

[45] Morris J (1993). The clinical dementia rating (CDR): Current version and scoring rules. Neurology.

[46] Rami L, Mollica MA, García-Sanchez C, Saldaña J, Sanchez B, Sala I (2014). The Subjective Cognitive Decline Questionnaire (SCD-Q): A validation study. J. Alzheimers Dis. JAD..

[47] Galvin JE, Roe CM, Powlishta KK, Coats MA, Muich SJ, Grant E (2005). The AD8: A brief informant interview to detect dementia. Neurology.

[48] Zigmond AS, Snaith RP (1983). The hospital anxiety and depression scale. Acta Psychiatr. Scand..

[49] Warrington EK (1996). The Camden Memory Tests.

[50] Wechsler D (1987). Manual for the Wechsler Memory Scale-Revised.

[51] Wechsler D (1999). Wechsler Abbreviated Scale of Intelligence (WASI) Manual.

[52] Stroop J (1935). Studies of interference in serial verbal reactions. J. Exp. Psychol..

[53] McKenna P, Warrington E (1983). The Graded Naming Test.

[54] Dunn, D. M. & Dunn, L. M. *The British Picture Vocabulary Scale*, 3rd ed. (2009).

[55] Jackson M, Warrington EK (1986). Arithmetic skills in patients with unilateral cerebral lesions. Cortex.

[56] Warrington EK, James M (1991). The Visual Object and Space Perception Battery.

[57] Wechsler D (1981). Wechsler Adult Intelligence Scale-Revised: Manual.

[58] Law R, O’Carroll RE (1998). A comparison of three measures of estimating premorbid intellectual level in dementia of the Alzheimer type. Int. J. Geriatr. Psychiatry..

[59] Nelson H (1991). National Adult Reading Test Manual.

[60] Shakespeare TJ, Kaski D, Yong KXX, Paterson RW, Slattery CF, Ryan NS (2015). Abnormalities of fixation, saccade and pursuit in posterior cortical atrophy. Brain.

