# Supplementary material for: Eye-tracking indices of impaired encoding of visual short-term memory in familial Alzheimer’s disease
Source: Sci Rep. 2021 Apr 22;11:8696. doi: 10.1038/s41598-021-88001-4 (PMC8062689; doi:10.1038/s41598-021-88001-4)
Supplement: Supplementary file 1 — Supplementary Information. [file 41598_2021_88001_MOESM1_ESM.pdf]

Supplementary Materials

Eye-tracking indices of impaired encoding of visual short-term memory in familial Alzheimer's disease

Ivanna M. Pavisic* [1,2], Yoni Pertzov [3], Jennifer M. Nicholas [1,4], Antoinette O'Connor [1,2], Kirsty Lu [1], Keir X.X. Yong [1], Masud Husain [5,6], Nick C. Fox [1,2], Sebastian J. Crutch [1,2]

1. Dementia Research Centre, Department of Neurodegenerative Diseases, UCL Queen Square Institute of Neurology, London, UK
2. UK Dementia Research Institute at University College London, London, UK
3. Department of Psychology, The Hebrew University of Jerusalem, Israel
4. Department of Medial Statistics, London School of Hygiene and Tropical Medicine, London, UK
5. Nuffield Department of Clinical Neuroscience, University of Oxford, UK
6. Department of Experimental Psychology, University of Oxford, UK

Here we provide supplementary information on Methods and Results including further details on visual exploration strategy metrics and findings on the nearest item control (NIC) and swap error proportion VSTM metrics.

Methods

Additional metrics of task performance calculated for 3-item conditions only

- Swap errors: In accordance with previous studies [1,2], a threshold of 4.5° was used as objects were never presented less than 9° from each other in the memory array and therefore an object could not be swapped with more than one object. (n=4 individuals with a score >2.5 SD were excluded from the analysis)
- Nearest item control (NIC): It is an index of localisation precision regardless of object identity. It provides a measure of localisation error discounting the effects of swap errors [1,3] n=1 individual with a score >2.5 SD were excluded from the analysis).

Similar to the localisation error metric, NIC was log-transformed prior to the linear regression analysis due to a skewed distribution. Analysis of swap errors used a logistic regression model. All models used robust standard errors to account for clustering by participant.

Results

Visual exploration strategies as predictors of VSTM performance: Identification accuracy and localisation error further details

In order to allow for comparison between models, Table S1, S2 and S3 excludes all participants with a score >2.5 SD in a visual exploration strategy metrics (n=2).

Table S1. Individual visual exploration strategies as predictors of VSTM performance.

	Adjusted by NART, sex, delay and:	**Adjusted mean [95% CI]<br>Group difference [95% CI] (control as reference)**<br>Controls	Early PMCs	Late PMCs	SMCs
Identification accuracy (% correct)<br>Odds Ratio for correct response	DT	88.5 [86.0, 91.1]<br>NA	88.1 [83.3, 92.9]<br>0.96 [0.56, 1.62]	87.1 [82.2, 92.1]<br>0.88 [0.53, 1.47]	77.4 [69.7, 85.0]<br>**0.43 [0.26, 0.73] ****
	Eq	88.3 [85.7, 91.0]<br>NA	88.6 [83.9, 93.3]<br>1.03 [0.60, 1.76]	87.4 [82.7, 92.1]<br>0.92 [0.55, 1.52]	77.2 [69.1, 85.3]<br>**0.44 [0.25, 0.76] ****
	S	88.6 [85.9, 91.3]<br>NA	88.6 [83.6, 93.6]<br>1.00 [0.56, 1.77]	87.8 [83.0, 92.5]<br>0.92 [0.55, 1.57]	76.0 [67.3, 84.8]<br>**0.40 [0.23, 0.70] ****
	Pr	88.7 [86.1, 91.4]<br>NA	88.9 [84.0, 93.6]<br>1.01 [0.57, 1.77]	87.6 [82.5, 92.7]<br>0.90 [0.52, 1.55]	75.3 [66.7, 83.8]<br>**0.38 [0.22, 0.65] ****
Localisation error (deg, log-transformed)<br>% difference	DT	1.63 [1.51, 1.74]<br>NA	1.66 [1.49, 1.83]<br>-4.00 [-15.10, 27.40]	1.68 [1.47, 1.90]<br>6.00 [-16.23, 34.10]	2.16 [1.97, 2.35]<br>**71.24 [37.56, 113.19] ****
	Eq	1.62 [1.50, 1.75]<br>NA	1.64 [1.46, 1.82]<br>1.92 [-17.87, 26.47]	1.67 [1.45, 1.89]<br>4.69 [17.84, 33.41]	2.20 [1.99, 2.40]<br>**77.32 [40.69, 123.50] ****
	S	1.61 [1.49, 1.73]<br>NA	1.63 [1.48, 1.78]<br>-2.11 [-15.97, 24.09]	1.67 [1.45, 1.90]<br>6.70 [-16.37, 36.15]	2.25 [2.05, 2.45]<br>**89.43 [50.05, 139.14] ****
	Pr	1.62 [1.49, 1.74]<br>NA	1.64 [1.47, 1.82]<br>-2.35 [-17.11, 26.39]	1.67 [1.44, 1.89]<br>5.01 [-17.86, 34.24]	2.23 [2.02, 2.43]<br>**84.02 [45.78, 132.29] ****

PMC=presymptomatic mutation carrier; SMC=symptomatic mutation carrier; CI=confidence intervals; DT=total dwell time on fractals; Eq=Equality; S=total number of shifts between fractals; Pr=proportion of time spent looking at the target. NA=not applicable. Bold=significant; *: significant at <0.05; **: significant at p <0.01.

Table S2. Individual visual exploration strategies as predictors of localisation performance by delay.

	**Adjusted by NART, sex, delay and:**	**Adjusted mean [95% CI] in deg (log-transformed) % group difference [95% CI] (control as reference)** Controls	Early PMCs	Late PMCs	SMCs
1-second	DT	1.51 [1.40, 1.63]<br>NA	1.51 [1.28, 1.74]<br>-0.38 [-22.14, 27.46]	1.58 [1.33, 1.83]<br>7.06 [-17.68, 39.25]	2.10 [1.90, 2.30]<br>**80.47 [42.84, 128.00] ****
	Eq	1.51 [1.39, 1.64]<br>NA	1.49 [1.25, 1.73]<br>-2.16 [-24.32, 26.49]	1.56 [1.31, 1.81]<br>5.11 [-20.00, 38.11]	2.14 [1.92, 2.36]<br>**87.58 [45.13, 142.45] ****
	S	1.50 [1.38, 1.63]<br>NA	1.48 [1.27, 1.70]<br>-1.73 [-22.76, 25.01]	1.57 [1.31, 1.82]<br>6.55 [-19.31, 40.69]	2.18 [1.97, 2.38]<br>**96.45 [52.80, 155.55] ****
	Pr	1.51 [1.38, 1.63]<br>NA	1.49 [1.26, 1.72]<br>-1.44 [-23.18, 26.44]	1.56 [1.30, 1.82]<br>5.72 [-19.80, 39.38]	2.17 [1.95, 2.38]<br>**93.61 [50.44, 149.17] ****
4-seconds	DT	1.74 [1.58, 1.90]<br>NA	1.82 [1.63, 2.01]<br>8.14 [-15.19, 37.90]	1.79 [1.60, 1.98]<br>5.03 [-16.88, 32.74]	2.22 [1.98, 2.47]<br>**61.91 [20.86, 116.90] ****
	Eq	1.74 [1.58, 1.91]<br>NA	1.80 [1.59, 2.00]<br>5.80 [-18.14, 36.73]	1.78 [1.59, 1.98]<br>4.29 [-17.99, 32.62]	2.25 [2.00, 2.50]<br>**66.92 [24.50, 123.80] ****
	S	1.72 [1.56, 1.88]<br>NA	1.78 [1.60, 1.96]<br>5.65 [-16.79, 34.13]	1.79 [1.59, 1.98]<br>6.82 [-15.73, 35.41]	2.32 [2.06, 2.59]<br>**82.64 [35.56, 146.06] ****
	Pr	1.73 [1.57, 1.90]<br>NA	1.79 [1.59, 1.99]<br>5.63 [-18.30, 36.56]	1.78 [1.58, 1.97]<br>4.11 [-18.36, 32.77]	2.29 [2.03, 2.55]<br>**74.45 [29.63, 134.77] ****

PMC=presymptomatic mutation carrier; SMC=symptomatic mutation carrier; CI=confidence intervals; DT=total dwell time on fractals; Eq=Equality; S=total number of shifts between fractals; Pr=proportion of time spent looking at the target. NA=not applicable. Bold=significant; *: significant at <0.05; **: significant at p <0.01.

Additional behavioural metrics of task performance: NIC and swap error proportion

Consistent with previous studies [2,4], irrespective of the group, performance for NIC and swap errors were also significantly influenced by delay (1s *vs* 4s).

In comparison to controls, SMCs had a significantly greater NIC error (49.71 [21.94, 83.80] %, p<0.001) and a significantly higher proportion of swaps (difference in square root of swap error: coefficient= 0.474 [0.006, 0.943] which equates to a generalised mean difference of 0.15 [0.001, 0.37] in swap proportion between groups, p=0.047). No significant differences emerged in PMCs (early: p=0.444 or late PMC: p=0.435) *vs* controls (**Fig.S1**).

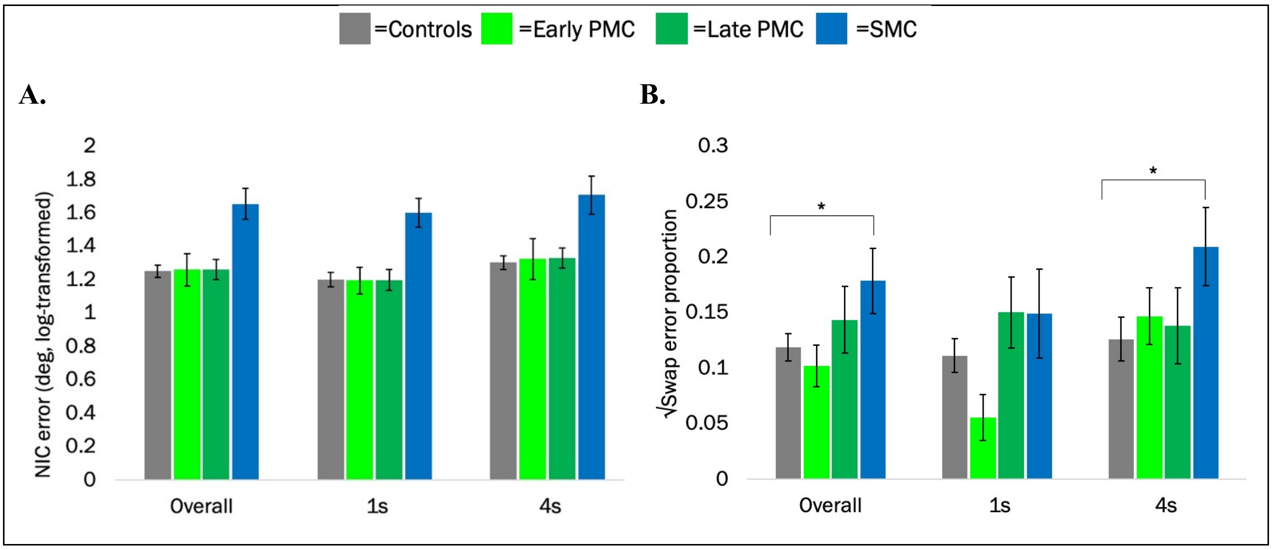

Figure S1. Behavioural VSTM performance by group. Adjusted mean performance (for age, sex and NART) by group. **A**. NIC overall and by delay (log-transformed). **B.** Swap error proportion overall and by delay (sqrt-transformed). Error bars represent standard errors of the group means. NIC=nearest item control; PMC=presymptomatic mutation carrier; SMC=symptomatic mutation carrier. Note that both metrics are measured in high memory load only (3-items trials). *: significant at *p*<0.05; **: significant at *p*<0.01.

Visual exploration strategies as predictors of NIC and swap error performance

NIC error:

Across the sample as a whole, increasing dwell time on fractals, equality score and time spent looking at the target (in proportion to the overall time spent on all fractals) were independently associated with a more accurate NIC performance. Across all groups, for every 100ms increase in the total dwell time on fractals for a given trial, NIC decreased by 1.21 [-0.02, 2.43] % error (*p*=0.054). Similarly, a higher Eq score resulted in a reduction of NIC error (*p*=0.008) in that trial. To put this into context, with Eq=0.5 *vs* Eq=1 NIC error (deg, log-transformed) resulted in 1.39 [1.32, 1.46] deg *vs* 1.25 [1.17, 1.33] deg, respectively. Increasing time spent looking at the target (in proportion to the overall time spent on all fractals), was also significantly associated with a reduction in NIC error (*p*=0.001). When excluding SMCs, associations with Pr remained (DT: *p*=0.293 and Eq: *p*=0.072; Pr: *p*<0.001). No significant association was observed for the total shifts between fractals (*p*=0.705).

There was no significant interaction between group, delay and the total dwell time on fractals (early PMCs: *p*=0.393; late PMCs: *p*=0.103, SMCs: *p*=0.442) nor between group, delay and the time spent fixating the target (early PMCs: *p*=0.272; late PMCs: *p*=0.632, SMCs: *p*=0.193). However, there was a trend for a greater reduction of NIC error in the 4-seconds compared to the 1-seconds delay condition, with increasing equality scores for SMCs compared to controls (*p*=0.063). To put this into context, with Eq=0.5 *vs* Eq=1: NIC error (log transformed) was 1.73 [1.54, 1.92] deg and 1.40 [1.22, 1.59] deg for SMCs and 1.27 [1.19, 1.34] deg and 1.24 [1.14, 1.34] deg for controls, though this did not reach

statistical significance. There was no significant interaction in Eq scores for the PMC groups (early: p=0.723; late: p=0.767).

Swap error proportion:

Across the sample as a whole, there was no evidence that the total dwell time on fractals (p=0.885), equality scores (p=0.863) or the proportion of time spent fixating the fractal (p=0.355) were associated with swap error performance.

There was some evidence that across the sample as a whole, a greater number of shifts resulted in a *greater* proportion of swaps. This trend ($\sqrt{OR}$=1.141 [0.997, 1.306], p=0.056) strengthened and reached significance when excluding SMCs ($\sqrt{OR}$=1.250 [1.308, 1.400], p=0.015). Interactions between group, delay and shifts were not significant (early PMCs: p=0.240; late PMCs: p=0.229; SMCs: p=0.959).

Considering visual exploration strategies as additional predictors of NIC and swap error performance, resulted in similar effect size differences between groups (Table S3).

Table S3. Visual exploration strategies as predictors of NIC and swap error performance (across all delays).

	Adjusted by NART, sex, delay and:	Adjusted mean [95% CI]<br>Group difference [95% CI] (control as reference)<br>Controls	Early PMCs	Late PMCs	SMCs
NIC error (deg, log-transformed) % difference		1.25 [1.18, 1.33]<br>NA	1.35 [1.21, 1.49]<br>10.00 [-5.40, 27.92]	1.27 [1.14, 1.39]<br>0.96 [-11.96, 15.78]	1.66 [1.47, 1.84]<br>**50.27 [23.15, 83.37]** **
	DT, Eq, S and Pr	1.26 [1.19, 1.33]<br>NA	1.36 [1.22, 1.49]<br>10.07 [-5.17, 27.76]	1.27 [1.16, 1.39]<br>1.58 [-11.24, 16.25]	1.62 [1.45, 1.78]<br>**42.94 [19.42, 71.09]** **
	DT	1.26 [1.19, 1.33]<br>NA	1.36 [1.22 1.50]<br>10.82 [-4.61, 28.75]	1.27 [1.16, 1.39]<br>1.43 [-11.17, 15.82]	1.62 [1.45, 1.79]<br>**42.99 [19.00, 71.82]** **
	Eq	1.26 [1.19, 1.33]<br>NA	1.35 [1.21, 1.49]<br>9.22 [-6.22, 27.19]	1.27 [1.15, 1.38]<br>0.46 [-11.99, 14.66]	1.63 [1.46, 1.80]<br>**44.87 [20.99, 73.47]** **
	S	1.25 [1.18,1.33]<br>NA	1.34 [1.21, 1.48]<br>9.64 [-5.70, 27.49]	1.26 [1.14, 1.39]<br>1.03 [-11.96, 15.94]	1.66 [1.48, 1.84]<br>**50.24 [24.18, 81.78]** **
	Pr	1.25 [1.18, 1.33]<br>NA	1.35 [1.21, 1.49]<br>9.64 [5.80, 27.62]	1.26 [1.14, 1.38]<br>0.73 [12.28, 15.68]	1.66 [1.47, 1.84]<br>**49.92 [22.98, 82.77]** **
Swap error: √proportion √ Odds Ratio		0.125 [0.101, 0.148]<br>NA	0.098 [0.058, 0.138]<br>0.763 [0.464, 0.125]	0.144 [0.086, 0.202]<br>1.184 [0.699, 2.003]	0.173 [0.113, 0.234]<br>1.478 [0.913, 2.393]
	DT, Eq, S and Pr	0.123 [0.101, 0.144]<br>NA	0.094 [0.056, 0.132]<br>0.740 [0.453, 1.208]	0.149 [0.093, 0.204]<br>1.254 [0.763, 2.062]	0.181 [0.116, 0.246]<br>1.596 [0.964, 2.644]
	DT	0.124 [0.101, 0.148]<br>NA	0.097 [0.058, 0.137]<br>0.757 [0.462, 1.242]	0.143 [0.086, 0.200]<br>1.179 [0.697, 1.994]	0.176 [0.110, 0.243]<br>1.513 [0.901, 2.543]
	Eq	0.125 [0.101, 0.148]<br>NA	0.098 [0.061, 0.136]<br>0.764 [0.478, 1.222]	0.159 [0.102, 0.215]<br>1.327 [0.818, 2.152]	0.180 [0.116, 0.243]<br>1.538 [0.932, 2.540]
	S	0.122 [0.100, 0.143]<br>NA	0.096 [0.058, 0.134]<br>0.763 [0.470, 1.239]	0.148 [0.091, 0.204]<br>1.254 [0.756, 2.081]	0.183 [0.123, 0.244]<br>**1.629 [1.023, 2.596]** *
	Pr	0.120 [0.095, 0.144]<br>NA	0.102 [0.068, 0.136]<br>0.762 [0.463, 1.254]	0.159 [0.102, 0.217]<br>1.119 [0.701, 2.009]	0.186 [0.127, 0.244]<br>1.476 [0.909, 2.399]

PMC=presymptomatic mutation carrier; SMC=symptomatic mutation carrier; CI=confidence intervals; NIC=nearest item control; DT=total dwell time on fractals; Eq=Equality; S=total number of shifts between fractals; Pr=proportion of time spent looking at the target. NA=not applicable. Bold: significant; *: significant at p <0.05; **: significant at p <0.01.

Further discussions on NIC error and swap proportion findings

NIC findings are in support of the weakening encoding hypothesis proposed for the localisation performance of late PMCs. The principal difference between localisation error and the NIC measures is that the latter accounts for the distance to the closest fractal as opposed to the correct fractal. Therefore, if we consider localisation and NIC measures in a continuum, localisation performance may account for correct binding and NIC for incorrect binding. In line with this, it is feasible that the stronger

reliance of the total dwell time on fractals was specific to the localisation error measure i.e. to the *binding* between the object's identity and location.

Lastly and contrary to some literature [1,5–7], no difference between PMC and controls was observed in the binary measure of swap errors (misbinding). While it is possible that our findings lack statistical power, unlike its behavioural counterparts, swap errors were not associated with many eye-tracking measures. This suggests that other mechanisms perhaps not accounted for here, may also explain swap error performance or that the binary nature of this metric made it less sensitive to detect subtle changes.

References

1. Liang Y., et al. Visual short-term memory binding deficit in familial Alzheimer's disease. *Cortex*. **78**, 150–164 (2016). doi:10.1016/j.cortex.2016.01.015

2. Pertzov Y., Dong M.Y., Peich M-C., Husain M. Forgetting What Was Where: The Fragility of Object-Location Binding. *PLOS ONE.* **7**, e48214 (2012). doi:10.1371/journal.pone.0048214

3. Pertzov Y., et al. Binding deficits in memory following medial temporal lobe damage in patients with voltage-gated potassium channel complex antibody-associated limbic encephalitis. *Brain*. **136**, 2474–2485 (2013). doi:10.1093/brain/awt129

4. Pertzov Y., Heider M., Liang Y., Husain M. Effects of healthy ageing on precision and binding of object location in visual short term memory. *Psychol Aging.* **30**, 26–35 (2015). doi:10.1037/a0038396

5. Parra M.A., Abrahams S., Logie R.H., Méndez L.G., Lopera F., Della Sala S. Visual short-term memory binding deficits in familial Alzheimer's disease. *Brain*. **133**, 2702–2713 (2010). doi:10.1093/brain/awq148

6. Parra M.A., Sala S.D., Abrahams S., Logie R.H., Méndez L.G., Lopera F. Specific deficit of colour-colour short-term memory binding in sporadic and familial Alzheimer's disease. *Neuropsychologia*. **49**, 1943–1952 (2011). doi:10.1016/j.neuropsychologia.2011.03.022

7. Parra M.A., et al. Memory binding and white matter integrity in familial Alzheimer's disease. *Brain*. **138**, 1355–1369 (2015). doi:10.1093/brain/awv048
